# Metastatic Anal Squamous Cell Carcinoma Presenting as an Indeterminate Biliary Stricture Diagnosed By Cholangioscopy

**DOI:** 10.14309/crj.0000000000000785

**Published:** 2022-06-23

**Authors:** Ritu Nahar, Ian Holmes, Jeffrey Baliff, Austin Chiang, Thomas Kowalski

**Affiliations:** 1 Department of Gastroenterology and Hepatology, Thomas Jefferson University Hospital, Philadelphia, PA; 2 Department of Pathology, Thomas Jefferson University Hospital, Philadelphia, PA

## Abstract

Anal squamous cell carcinoma (SCC) rarely metastasizes outside the pelvis. Although liver involvement has been described, biliary strictures from metastatic disease are exceedingly rare. We report a case of a patient with metastatic anal SCC presenting as a biliary stricture, which was identified on endoscopic retrograde cholangiopancreatography with single-operator cholangioscopy. Direct visualization of the stricture with single-operator cholangioscopy may prove critical in obtaining a timely diagnosis. Therapeutic options for metastatic anal SCC are limited, but chemotherapy can be considered, and surgical resection is an option for limited disease.

## INTRODUCTION

Anal squamous cell carcinoma (SCC) rarely metastasizes outside the pelvis.^1^ When extrapelvic disease is identified, liver metastases may be seen, but biliary strictures are exceedingly rare.^2^ Metastatic anal SCC presenting as a biliary stricture has only been described once before in the literature, where the diagnosis was made by exploratory laparoscopy.^3^ In this study, we present a case of metastatic anal SCC presenting as an indeterminate biliary stricture, where the diagnosis was ultimately determined by endoscopic retrograde cholangiopancreatography (ERCP) with single-operator cholangioscopy.

## CASE REPORT

**Figure 1.**
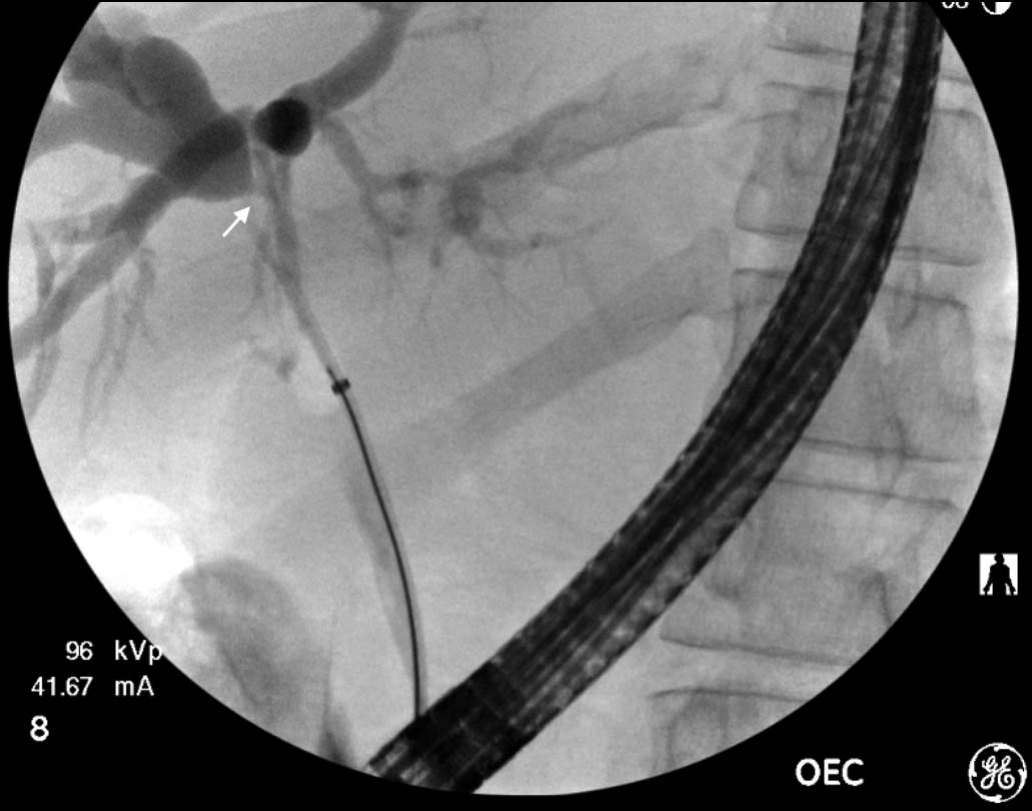
Fluoroscopy from previous endoscopic retrograde cholangiopancreatography demonstrating a hilar stricture with proximal dilation of the intrahepatic biliary tree.

**Figure 2.**
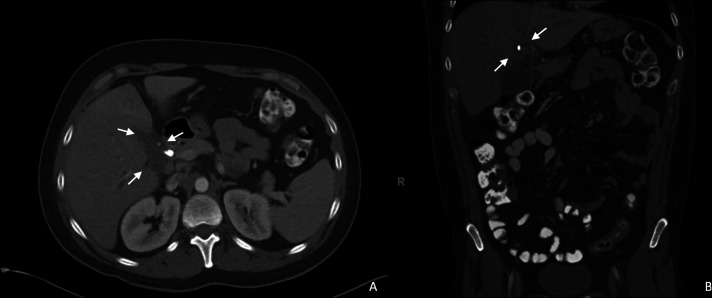
A CT scan of the abdomen and pelvis before endoscopic retrograde cholangiopancreatography. (A) Axial image demonstrating 5.3 by 3.5 by 3.2 cm ill-defined hypoattenuating lesion at the porta hepatis. (B) Coronal image demonstrating intrahepatic biliary ductal dilation proximal to the lesion.

A 31-year-old male patient presented with abdominal pain and jaundice. The patient's history was notable for HIV well controlled with elvitegravir, cobicistat, emtricitabine, and tenofovir. His past HIV viral load had been 26.2 copies/mL. He had anal squamous cell carcinoma, which had been diagnosed 1 year previously and had been treated with 5-fluorouracil, radiation, and a diverting sigmoid colostomy. He had previously presented to an outside hospital for jaundice and fatigue, with a total bilirubin of > 30 mg/dL. An abdominopelvic computed tomography (CT) scan showed new intrahepatic biliary ductal dilation at the level of the porta hepatis without extrahepatic ductal dilation. He had undergone an endoscopic ultrasound and ERCP for a hilar biliary stricture at that time, but the mass was not accessible for fine-needle biopsy, cytologic brushings and bile aspiration were nondiagnostic, and he was lost to follow-up (Figure 1). His laboratory evaluations were notable for an aspartate aminotransferase of 112 IU/L, an alanine aminotransferase of 115 IU/L, an alkaline phosphatase of 985 IU/L, a total bilirubin of 14.0 mg/dL, and a direct bilirubin of 13.2 mg/dL. An abdominopelvic CT scan demonstrated a new left portal vein thrombosis and an ill-defined, hypoattenuating lesion at the portal confluence measuring 5.3 by 3.5 by 3.2 cm (Figure 2). Positron emission tomography (PET CT) did not demonstrate other metastatic lesions.

**Figure 3.**
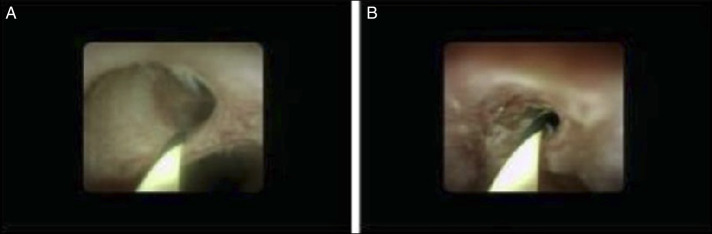
Cholangioscopy images from endoscopic retrograde cholangiopancreatography. (A) An abnormal vascular pattern noted at the level of the stricture. (B) Sclerosis and edema noted at the level of the stricture.

**Figure 4.**
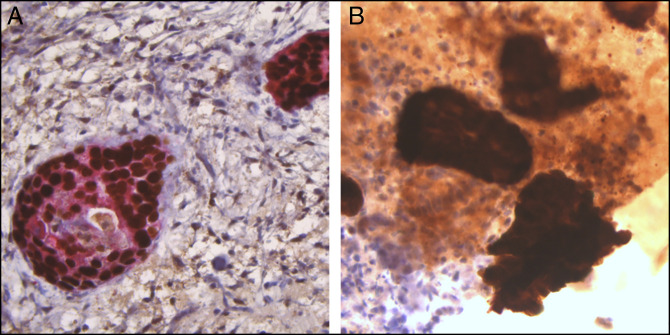
Pathology from endoscopic retrograde cholangiopancreatography biopsies. (A) Double immunohistochemical stain at ×400 magnification for p40 (nuclear, brown) and CK5/6 (cytoplasmic, red) supports squamous differentiation. (B) Immunohistochemical stain for p16 (a surrogate marker for human papillomavirus infection) at ×400 magnification is strongly positive, supporting metastasis from the patient's known anal squamous cell carcinoma.

An ERCP was performed. Single-operator cholangioscopy revealed a sclerotic-appearing stricture at the level of the proximal common hepatic duct with areas of edema and abnormal vascularity (Figure 3). Cholangioscopically directed biopsies of the stricture were performed, and cholangiographically directed biopsies and cytologic brushings were also collected. Plastic stents were placed in the left hepatic duct and the right anterior sectoral duct. Cholangioscopically directed and cholangiographically directed biopsies showed tumor cells positive for p40, CK5/6, and CK19 but negative for synaptophysin, consistent with squamous cell carcinoma. Rare tumor cell clusters were also positive for p16, a surrogate marker for human papillomavirus infection, suggesting that the biliary stricture was secondary to a metastasis from the patient's anal SCC. (Figure 4).

## DISCUSSION

Indeterminate biliary strictures are a common diagnostic dilemma for the pancreatobiliary endoscopist. Sampling methods for biliary strictures vary widely in sensitivity and specificity. This patient underwent bile aspiration, cytologic brushings, cholangiographically directed biopsies, cholangioscopically directed biopsies, and EUS. Bile aspiration can have a sensitivity rate as low as 5%–30%. Cytologic brushings can have a sensitivity as low as 35%, although specificity is usually >90%. Cholangiographically directed biopsies have a sensitivity around 45%, although specificity is usually >90%. Endoscopic ultrasound-guided fine needle biopsy can have a sensitivity of >70%, although there is a theoretical risk of seeding tumor cells along the tract of the needle, so it may not always be an option if a distinct mass is not present or if surgical resection is possible.^4^ Finally, cholangioscopically directed biopsies are performed under direct visualization using a choledochoscope passed through the duodenoscope into the biliary tract. A recent multinational registry reported that biopsies performed in this manner were 75.3% sensitive and 100% specific and also allowed for a visual diagnostic impression of the stricture in 87.2% of the patients.^5^ In this patient, cholangioscopy proved to be the key difference maker in identifying squamous cell carcinoma of the bile duct.

SCC of the bile duct is exceedingly rare, with an incidence of 0.2% of all biliary tract malignancies.^6^ Primary SCC of the bile duct can occur from hepatolithiasis, recurrent pyogenic cholangitis, or Clonorchis infection causing squamous metaplasia. Other possibilities include squamous metaplasia of preexisting adenocarcinoma or heterotopic squamous epithelium with malignant transformation.^7^ Primary SCC can also arise because of chronic inflammation within choledochal cysts.^8–10^ However, the positive p16 staining of this patient's tumor cells suggests that his biliary stricture was not due to primary SCC but was in fact due to metastatic SCC.

Anal SCC is most commonly identified when it is still confined to the pelvis. According to the Surveillance, Epidemiology and End Results (SEER) database, 50% of anal SCCs are localized at the time of initial diagnosis while 29% spread to regional lymph nodes and 12% have distant metastases. These presentations carry a 5-year survival of 80%, 60% and 30.5% respectively.^1^ The most common sites of extrapelvic involvement are the liver, lung, and extrapelvic lymph nodes. Given that anal SCC is an uncommon tumor and extrapelvic metastatic disease occurs in 10%–20% of these patients, limited data are available on this population.^2^ Metastases to rare sites such as the duodenum and cranial bones have also been described.^11,12^ Human papillomavirus DNA load and p16 expression have also been suggested to predict the survival of patients with localized anal SCC treated with chemoradiotherapy.^13^

To the best of our knowledge, this is the second case of metastatic anal SCC presenting as a biliary stricture to be described in the literature. Bass et al previously described a 55-year-old female patient with a history of moderately differentiated anal SCC who had presented with 1 week of jaundice and epigastric pain. An abdominopelvic CT scan had shown marked intrahepatic and extrahepatic biliary ductal dilation with an ill-defined, hypodense 3.0 by 2.7 cm porta hepatis mass. The mass was successfully surgically resected, and a Roux-en-Y hepaticojejunostomy was performed. The tumor stained positive for p63 and CK5/6 but negative for CK20 and CDX2, consistent with SCC.^3^ However, their patient did not undergo ERCP or cholangioscopy before resection. In the patient in this report, the edema and altered vascularity of the stricture as visualized on cholangioscopy, as well as the cholangioscopically directed biopsies, proved invaluable in his ultimate diagnosis.

## DISCLOSURES

Author contributions: I. Holmes is the article guarantor. R. Nahar and I. Holmes reviewed the literature and contributed to the article's drafting. J. Baliff provided pathology slides and interpretation. A. Chiang and T. Kowalski analyzed the imaging findings and were responsible for revision of the article for important intellectual content. All authors issued final approval for the version to be submitted.

Financial disclosure: A. Chiang: Consultant—Boston Scientific. T. Kowalski: Consultant—Olympus, Boston Scientific, and Medtronic. The authors have no competing interests.

Informed consent was obtained for this case report.
